# The taxonomist - an endangered race. A practical proposal for its survival

**DOI:** 10.1186/1742-9994-8-25

**Published:** 2011-10-26

**Authors:** Heike Wägele, Annette Klussmann-Kolb, Michael Kuhlmann, Gerhard Haszprunar, David Lindberg, André Koch, J Wolfgang Wägele

**Affiliations:** 1 Zoologisches Forschungsmuseum Alexander Koenig, Adenauerallee 160, 53113 Bonn, Germany; 2 Institute for Ecology, Evolution and Diversity, Goethe-University, Siesmayerstrasse 70, 60054 Frankfurt am Main, Germany; 3 Department of Entomology, The Natural History Museum, Cromwell Road, London SW7 5BD, UK; 4 Zoologische Staatssammlung München, Münchhausenstraße 21, 81247 München, Germany; 5 Department of Integrative Biology and Museum of Paleontology, University of California, Berkeley, CA 94720-4780, USA

**Keywords:** Taxonomy crisis, taxonomic impediment, impact factor, original species description, citation index, systematics

## Abstract

**Background:**

Taxonomy or biological systematics is *the *basic scientific discipline of biology, postulating hypotheses of identity and relationships, on which all other natural sciences dealing with organisms relies. However, the scientific contributions of taxonomists have been largely neglected when using species names in scientific publications by not citing the authority on which they are based.

**Discussion:**

Consequences of this neglect is reduced recognition of the importance of taxonomy, which in turn results in diminished funding, lower interest from journals in publishing taxonomic research, and a reduced number of young scientists entering the field. This has lead to the so-called taxonomic impediment at a time when biodiversity studies are of critical importance.

Here we emphasize a practical and obvious solution to this dilemma. We propose that whenever a species name is used, the author(s) of the species hypothesis be included and the original literature source cited, including taxonomic revisions and identification literature - nothing more than what is done for every other hypothesis or assumption included in a scientific publication. In addition, we postulate that journals primarily publishing taxonomic studies should be indexed in ISI^SM^.

**Summary:**

The proposal outlined above would make visible the true contribution of taxonomists within the scientific community, and would provide a more accurate assessment for funding agencies impact and importance of taxonomy, and help in the recruitment of young scientists into the field, thus helping to alleviate the taxonomic impediment. In addition, it would also make much of the biological literature more robust by reducing or alleviating taxonomic uncertainty.

## Background

Taxonomy or biological systematics is the science of discovering, describing, classifying and naming organisms [1]. It dates from Aristotle, and thus is the oldest discipline in biology. It forms the basis for all other scientific disciplines dealing with the study of life, its structure, function and evolution. Taxonomic knowledge is paramount whether studying whole organisms, their organs, their specific bio-molecules or biochemical pathways. Unambiguously identified organisms in the sense of referenced, unique taxonomy are essential to all biological studies, because they are a prerequisite to enable the confirmation or refutation of any scientific study that reports on these taxa or their components. Only if a species' identity is unambiguous, can the study be considered robust enough to discuss species-specific traits or attributes with colleagues or convey them to the scientific community and general public. Identification of a species utilized in a study impacts all subsequent comparisons, predictions, and possible replication of the study. It might be argued that the identification of the study organism is irrelevant when examining basic processes such as membrane transport mechanisms, photosynthetic cycles in plants, etc. However, this is not the case as many pathways of physiological processes are adapted in the organism to the specific environmental conditions and this must be taken into consideration whenever conclusions are generalized beyond the study organism. For example, the eubacterium *Thermus aquaticus *Brock & Freeze, 1969 [2] is restricted to hot springs and is the only species known (and famous for) possessing the *Taq*-Polymerase [3]. In addition, medical studies reporting on the leech *Hirudo medicinalis *Linnaeus, 1758 [4] have been shown to have actually studied several distinct yet morphologically cryptic leech species [5,6]. Given the importance of this species in neurobiology and the study of anticoagulants, it is critical that workers associate leech identifications with a referenced, unique taxonomy rather than use a non-specific, general species name. The identification of the species is the first step in almost any biological or related study.

In many cases species identifications are difficult, and it is not surprising that a staggering number of species remain undescribed [7-10]. Add to this the scarcity of knowledgeable taxonomists [11] (particularly in third world and developing countries [12]), often referred to as the "taxonomic impediment" [1,13-16], and it is understandable why certain biological disciplines have chosen "taxonomy-free" research subjects. In particular, many biodiversity and ecological research programs pursue directions that do not require estimates of the actual (alpha) diversity of ecosystems. Instead proxies such as biomass production, measurements of evaporation, CO_2 _storage, functional groups, or the focusing on a few selected and well-known taxonomic groups (such as "birds", "bats", or "trees') are substitute for measurements of alpha biodiversity [17]. In addition, funding to include alpha diversity studies across a wide variety of groups making up the community or occurring in the habitat is seldom allocated or made available.

Taxonomy is *the *discipline in biology where scientists assign to taxa unique identities, and these research products are subsequently used by others to identify further individuals that can be used with confidence by colleagues in other scientific disciplines. Many of these other disciplines often include investigations of smaller components of diversity, such as proteins and genes and larger cell components, but they also include population studies, habitat characterization, environmental monitoring and systemic modelling; investigations that include all types of organisms, from bacteria and protists to vertebrates and plants - the entire Tree of Life. The potential consequences of flawed taxonomy leading to error-cascades affecting scientific hypotheses and ideas are commonly underestimated or ignored, but may have serious ecological and economic implications [18].

The availability of species identifications for life science studies is often taken for granted. Currently, about 1.7 Mio metazoan species have been described and it is assumed that tenfold as many species inhabit our world [8,10,19,20] (but see Castello et al. 2011[21]). Studies of biodiversity are critical [10] and have been mandated by many countries where conservation and sustainable use of natural resources have become matters of scientific and public concern [22-24]. Taxonomy is fundamentally important in ensuring the quality of life of future human generations on the planet.

## Discussion

There is no question that taxonomists provide insights into alpha biodiversity, provide names for communication, and are at the forefront documenting the biological richness of our planet. Consequently, they must be recognized for their contributions and should be considered an important resource within biology and the associated life science, as well as by the general public. Moreover, journals and other publication media that convey this taxonomic knowledge should be considered as valuable as journals dedicated to other scientific subjects and findings. However, today we are faced with exactly the opposite scenario. Despite the increasing importance of taxonomists in today's biodiversity crisis, most taxonomists are faced with decreasing funding, as well as editorial resistance to publishing their work in the high impact journals that will secure tenure and promotion and allow them to continue their work and contributions [18,25,26].

Taxonomists are often looked upon by colleagues as bureaucratic accountants and their research programs are not recognized as the intellectually challenging and hypotheses-driven science that it has become [27-29]. Today's taxonomist must have a thorough knowledge of the literature, of theoretical species concepts, phylogenetic and analytical methodology, the application of various phenotypic visualization techniques (e.g., anatomy, histology, fine-structure, imaging, 3-D reconstruction), molecular staining, as well as molecular markers for everything from barcoding to genomics. When taxonomic hypotheses are implemented as published descriptions they are subject to future revisions (i.e., replication) with the possibility of either confirmation or rejection just as any other scientific hypothesis [29]. All this notwithstanding, the work done by the species' author(s) is rarely accredited [26,30,31]. Sometimes the author's name is included with the scientific name, but this citation is rarely included in the publication's references or literature cited. The genetic model organisms *Drosophila melanogaster *Meigen, 1830 [32] and *Arabidopsis thaliana *(L.) Heynh. [33], for instance, are among the most prominent species cited in scientific publications (about 352.000 and 232.000 citations, respectively). In sharp contrast, the original scientific descriptions of both these species are cited 0 and 19 times, respectively. Other frequently studied and prominent examples, including more recently described species, are presented in Table 1.

**Table 1:** Discrepancies between the use of species names in scientific publications and citations of the original authors.

Scientific species name, original author, number of citation	Common species name	Google scholar hits of publications using the species name	Google scholar hits of publications citing the original author(s) and description	**ISI**^SM ^**web of knowledge hits of publications citing the original author(s) and description**
**Model organisms**				

*Escherichia coli *(Migula, 1895) [39]	*E. coli*	ca. 1.640.000	ca. 58	-

*Arabidopsis thaliana *(Linnaeus, 1763)^a ^[33]	Mouse-ear cress	ca. 232.000	19	-

*Drosophila melanogaster *Meigen, 1830 [32]	Fruit fly	ca. 352.000	ca. 200	-

*Caenorhabditis elegans *(Maupas, 1899) [58]	-	ca. 173.000	52	-

*Mus musculus *Linnaeus, 1758 [4]	House mouse	ca. 108.000	ca. 300*	-

*Trichoplax adhaerens *Schulze, 1883 [59]	*-*	ca. 719	ca. 70	-

*Lycopoidoides moellendorffii *(Hieronymus, 1902)^a ^[60]	Spikemoss (*Selaginella moellendorffii*)	ca. 560	155**	-

*Amphimedon queenslandica *Hooper & van Soest, 2006 [61]	Sponge	ca. 335	9	7

*Macrostomum lignano *Ladurner et al., 2005 [62]	Flatworm	ca. 150	25	34

**Invasive species**				

*Batrachochytrium dendrobatidis *Longcore et al., 1999 [63]	Chytrid fungus	ca. 2130	317	246

*Boiga irregularis *(Bechstein, 1802) [64]	Brown tree snake	ca. 1760	1	-

*Eleutherodactylus coqui *Thomas, 1966 [65]	Common Puerto Rican Coqui frog	ca. 1510	16	-

*Cameraria ohridella *Deschka & Dimic, 1986 [66]	Horse-chestnut leaf miner	ca. 1320	ca. 30	Journal not indexed

**Prominent species**				

*Tyrannosaurus rex *Osborn, 1905 [67]	*T. rex*	ca. 5410	ca. 30	-

*Metasequoia glyptostroboides *Hu & Cheng, 1948 [68]	Dawn redwood	ca. 2510	53/58	-

*Latimeria chalumnae *Smith, 1939 [69]	West Indian Ocean coelacanth	ca. 2070	325	-

*Homo floresiensis *Brown et al. 2004 [70]	Flores man, nicknamed "hobbit"	ca. 1400	245	244

*Varanus komodoensis *Ouwens, 1912 [71]	Komodo dragon	ca. 1010	10	-

**Recently described species**				

*Euperipatoides kanangrensis *Reid, 1996 [72]	Onychophora	1800	33	47

*Cryptocorynetes haptodiscus *Yager, 1987 [73]	Remipedia	296	121	24

*Latimeria menadoensis *Pouyaud, 1999 [74]	Indonesian coelacanth	ca. 235	11	23

We compared hit results of online queries for species names used in publications listed by Google scholar and ISI^SM ^web of knowledge and those publications citing the original authors and descriptions. Full citations are given in the references section of this paper. Note that ISI^SM ^web includes only hits after 1945.

^a ^We have altered the abbreviated botanical format of the original author into the zoological format, which provides the author name in full length with the year of publication.

* Citations refer to the different spellings of the author's name (i.e., Linné, von Linné, and Linnaeus) and the entire tenth edition.

** Citations refer to the whole series "Die Natürlichen Pflanzenfamilien (...)", which was published between 1887 and 1909 in numerous volumes.

Subsequent taxonomic revisions that consolidate taxa (i.e., synonymization) or split species into different subclades are also usually neglected. Out of the 2270 citations found on *Hirudo medicinalis *in Google scholar for the year 2010, only 41 mentioned the presence of cryptic species and former misidentifications of *H. medicinalis *and *H. verbena *Carena, 1820 [34]. Hence, more than 95% of the analyses published in 2010 that explicitly dealt with *H. medicinalis *(covering a broad array of subjects, like genomes, proteomes, gene syntheses, medical novelties, etc.) cannot be clearly assigned to this species, nor to either of the two undescribed species within the *H. medicinalis *species complex [5,6], nor to the frequently misidentified *H. verbena*. Citing the original publication in which the cryptic species problem had been unravelled would have immediately demonstrated the authors' awareness of this problem and its potential consequences, and would have increased confidence in the author's publication.

Recently, several declarations and suggestions have been published in favour of taxonomy, including arguments for more funding, better education, or recruiting parataxonomists and amateurs [11,16,35]. While these appeals are certainly justified, they will not be effective as long as the work of taxonomists is neglected or viewed as unimportant and self-evident (and therefore not worthy of citation) by colleagues in other natural science disciplines. We therefore here emphasize the need for a fair practice mentioned by Werner in 2006 [30] and Seifert et al. in 2008 [36] that would help to recognize the value of taxonomic work and thus to place taxonomy back into mainstream biology and provide a measure of its impact. This accurate accounting of the value of taxonomic studies will also provide familiar metrics for colleagues and administrators, and will be invaluable in the allocation of funding and the long term recruitment of young taxonomists. We therefore propose the following guidelines:

1. Any study based on a formally named organism should include the citation of the original author(s) and date. We acknowledge that this is already practiced in many, but by no means all journals. In addition, this citation must be included in the literature cited - a practice that is currently extremely rare (e.g., *Peersonia; Phytotaxa; Blumea; Organisms, Diversity & Evolution *[37], *European Journal of Taxonomy*) or not obligatory, but encouraged (e.g., *Zootaxa*).

2. All published taxonomic sources (monographs, identification keys, primary taxonomic literature and revisions) used for identification or as a source of nomenclatural information (e.g., catalogues) should be cited as any other methodological paper would be. The lack of these citations precludes an assessment of quality and reliability of the identification(s) and associated taxonomic information. Thus, independent verification of results and conclusions - the fundament of science - is not possible.

3. Researchers are encouraged to include taxonomists as co-authors when they have made substantial contributions to the research program or where the conclusions of the paper are solely dependent on the accurate identification of the study taxon.

One of the leading journals in ecology, Ecology Letters, follows the proposed guidelines, with the exception of well-established species such as *Homo sapiens *Linnaeus, 1758 [38], *Drosophila melanogaster *and *Escherichia coli *(Migula, 1895) [39]. A most recent suggestion [40] dealt with a solution for special citation of taxonomic work when used in wiki pages by combining both the original non-wiki source and the respective wiki page.

It might be argued that publications that are based on studies of multiple species, such as large phylogenetic analyses of an entire metazoan or plant group (e.g., a phylogenetic analysis of beetles) or monitoring projects, might lead to an inflation of certain citations or journals [30,37]. However, this is no different than the long lists of GenBank entries for sequences or alignments that have come to dominate our publications and associated supplementary materials. Also, arguments that it is difficult to deal with the older literature are becoming increasingly obsolete as the number of online taxonomic databases (e.g., BHL, EoL, Gallica, AnimalBase, and others) rapidly increases. Authors working on projects involving large species data sets would also be more likely to seek out taxonomists for assistance with subsequent co-authorship - already a common practice for bioinformatical or mathematical problems in phylogenetic or statistical analyses. This will increase communication and collaboration as well as the accuracy and usefulness of the vetted work.

It might also be argued that citing old literature is not necessary and that taxonomic hypotheses should be handled as in other disciplines: these hypotheses become "general knowledge" and no longer require citation, such as the seminal discoveries of natural selection published by Wallace in 1855 [41] and Darwin in 1859 [42], or of plate tectonics published by Wegener in 1912 [43]. However, as was shown with the example of *Hirudo medicinalis*, even "well-established" species can become imprecise, and refined hypotheses with new species names must be formulated. Especially in the time of molecular analyses, we can expect many more surprises with broad implications for various fields, including human welfare. The recent findings [44] of unknown subgroups within the *Anopheles gambiae *Giles, 1902 [45] complex [46] that exhibit a high susceptibility to infection with wild *Plasmodium falciparum *(Welch, 1897) [47] must be cited in future analyses to acknowledge one's awareness of sympatric species with different ecological and behavioral strategies within the same strains. The finding of cryptic species in spitting cobras [48], as well as the rearrangement of the large species complex of the Asian pitvipers into distinct genera [49] has a direct implication on categorization of their medical importance, as well as antivenin indication, prescription and research [50]. Results like these are not restricted to small and cryptic living species but also comprise large animals such as turtles, monitor lizards, antelopes or bovids [51-53] with direct implications on conservation biology and related fields [53,54]. Hence it is more important than ever to include all means used for identification, so that the authors' awareness of taxonomic problems that can potentially confound their study, including species concepts applied [10], is obvious to the readers of their published results.

In addition, we would encourage all taxonomists, who are in one way or another responsible for journal administration to ensure that their publications are indexed by ISI^SM^. Based on the situation in molluscan literature (pers. comm. P. Mikkelsen), we assume that more than 90 percent of all taxonomic journals are not indexed so that the overwhelming numbers of taxonomic citations are simply not counted. The inclusion in the ISI^SM ^data base will increase the awareness of the journal and guarantee a more accurate calculation of journal and author citation metrics. We estimate that indexing half of all taxonomic journals available today would increase citation indices (CIs) fivefold.

## Summary

Taxonomic work has profound implications for all kinds of scientific disciplines. Previous attempts of a few colleagues to encourage citation policies concerning taxonomic descriptions [30,36] have been largely ignored. Therefore, we once again emphatically appeal to colleagues and editors of journals for a much broader acknowledgement of the scientific work of taxonomists. The citation protocol as outlined above would give fair credit and recognition to those scientists who have dedicated their research careers to unveiling the earth's biodiversity and to the journals who have specialized in reporting these results. Currently, neither is recognized for their important contributions irrespective of their geographical location [1]. In addition, citation of species and taxon authorities will validate the taxonomic names used in scientific studies and will increase the robustness and usefulness of their results.

We are well aware of the severe shortcomings and weaknesses of CIs in systematics and taxonomy [16,26,55-57]. However, we cannot ignore the system and its impact; instead we should fully participate to ensure fair and accurate representation of our colleagues and journals. The citation protocol outlined above will require little additional investment by researchers and editors, but would be an important acknowledgement of the vital contributions of taxonomists and hopefully increase the survival rate of this endangered group of scientists.

## Competing interests

The authors declare that they have no competing interests.

## Authors' contributions

HW initiated and drafted the manuscript, all other authors contributed equally to the manuscript. All authors read and approved the final manuscript.

## Author information

^1^Zoologisches Forschungsmuseum Alexander Koenig, Adenauerallee 160, 53113 Bonn, Germany

^2^Institute for Ecology, Evolution and Diversity, Goethe-University, Siesmayerstrasse 70, 60054 Frankfurt am Main, Germany

^3^Department of Entomology, The Natural History Museum, Cromwell Road, London SW7 5BD, UK

^4^Zoologische Staatssammlung München, Münchhausenstraße 21, 81247 München, Germany

^5^Department of Integrative Biology and Museum of Paleontology, University of California, Berkeley, CA 94720-4780, USA
